# Supplementary material for: SPA: A Probabilistic Algorithm for Spliced Alignment
Source: PLoS Genet. 2006 Apr 28;2(4):e24. doi: 10.1371/journal.pgen.0020024 (PMC1449883; doi:10.1371/journal.pgen.0020024)
Supplement: Table S1 — (37 KB PDF) [file pgen.0020024.st001.pdf]

Supporting Table S1: Statistics of the Mappings of Human full-length cDNAs

Erik van Nimwegen[a] Nicodeme Paul[a] Robert Sheridan[b]
Mihaela Zavolan[a]
[a]Biozentrum, the University of Basel, Switzerland
[b]current address: Memorial Sloan-Kettering Cancer Center, New York, USA

January 13, 2006

Statistic	SPA	Sim4	GMAP	BLAT	Spidey
Matched nucleotides	49,347,174 (99.1%)	49,241,404 (98.8%)	49,191,741 (98.7%)	49,175,779 (98.7%)	48,319,274 (97.0%)
Unmapped cDNAs	7	11 (+57%)	72 (+928%)	40 (+471%)	287 (+4000%)
Nucleotides in unmapped cDNAs	17,965	22,827 (+27%)	163,540 (+810%)	85,096 (+373%)	738,727 (+4012%)
Unmapped nucleotides at 5' end	118,050	142,901 (+21%)	154,095 (+31%)	146,996 (+25%)	316,663 (+168%)
Unmapped nucleotides at 3' end	118,275	150,151 (+27%)	169,039 (+42%)	173,907 (+47%)	157,899 (+34%)
Nucleotides in polyA tails	27,727	5409 (−80%)	28,762 (+4%)	33,237 (+20%)	28,437 (+3%)
Mismatched nucleotides	96,503	101,062 (+5%)	94,747 (−2%)	85,677 (−11%)	109,720 (+14%)
Inserted nucleotides	93,872	155,812 (+66%)	17,642 (−81%)	118,874 (+27%)	148,846 (+59%)
Deleted nucleotides	21,215	19,096 (−10%)	21,884 (+3%)	17,936 (−15%)	11,854 (−44%)
Splice boundary errors	7,316	20,813 (+184%)	6,179 (−16%)	7,663 (+5%)	24,611 (+236%)
Misoriented cDNAs	61 (0.3%)	-	218 (1.1%)	-	317 (1.6%)

Comparison of mapping statistics for SPA, Sim4, Gmap, Blat, and Spidey on a dataset of 20,207 human full-length cDNAs. The first row shows the total number of nucleotides mapped to matching nucleotides in the genome. The second row gives the number of cDNAs for which the algorithm produced no mapping or (for Gmap and Spidey) produced a corrupt output file. The third row gives the total number of nucleotides corresponding to the unmapped cDNAs. The fourth row shows the number of nucleotides in unmapped 5' ends, and row five the number of nucleotides in unmapped 3' ends. We consider any unmapped 3' end that consists of more than 80% As as a poly-A tail. Row six shows the total number of bases in poly-A tails so defined. Row seven shows the total number of nucleotides mapped to mismatching nucleotides in the genome. Row eight shows the total number of inserted nucleotides, defined as unmapped nucleotides that are internal to the mapping. Row nine shows the total number of nucleotides in deletions (genomic nucleotides missing from the clone that do not correspond to introns). Row ten shows the total number of nucleotides in insertions, deletions, or mismatches within 10 basepairs of the splice boundaries. Finally row eleven shows the total number of clones that were considered misoriented. In rows 1 and 11 the percentages indicate the fraction of the total number of nucleotides in all cDNAs, and the total number of cDNAs respectively. In all other rows the percentages indicate the relative change compared to the SPA mappings.
