# Supplementary material for: SPA: A Probabilistic Algorithm for Spliced Alignment
Source: PLoS Genet. 2006 Apr 28;2(4):e24. doi: 10.1371/journal.pgen.0020024 (PMC1449883; doi:10.1371/journal.pgen.0020024)
Supplement: Table S2 — (36 KB PDF) [file pgen.0020024.st002.pdf]

Supporting Table S2: Statistics of the Mappings of Mouse full-length cDNAs

Erik van Nimwegen[a] Nicodeme Paul[a] Robert Sheridan[b]
Mihaela Zavolan[a]
[a]Biozentrum, the University of Basel, Switzerland
[b]current address: Memorial Sloan-Kettering Cancer Center, New York, USA

January 13, 2006

Statistic	SPA	BLAT (post-processed)
Matched nucleotides	217,879,391 (98.8%)	215,727,288 (97.8%)
Unmapped cDNAs	215	656 (+205%)
Nucleotides in unmapped cDNAs	424,211	1,116,955 (+163%)
Unmapped nucleotides at 5' end	759,473	1,249,434 (+64.5%)
Unmapped nucleotides at 3' end	497,787	1,248,281 (+151%)
Nucleotides in polyA tails	2,463	0 (−100%)
Mismatched nucleotides	361,137	244,210 (−32.4%)
Inserted nucleotides	680,363	1,018,657 (+49.7%)
Deleted nucleotides	99,087	53,710 (−45.8%)
Splice boundary errors	24,621	40,287 (+63.6%)
Misoriented cDNAs	244 (0.24%)	-

Comparison of mapping statistics of the SPA and post-processed BLAT mappings for the RIKEN FANTOM3 dataset of $102,792$ full-length cDNAs. The first row shows the total number of nucleotides mapped to matching nucleotides in the genome. The second row gives the number of cDNAs for which the algorithm produced no mapping. The third row gives the total number of nucleotides corresponding to the unmapped cDNAs. The fourth row shows the number of nucleotides in unmapped 5' ends, and row five the number of nucleotides in unmapped 3' ends. We consider any unmapped 3' end that consists of more than 80% As as a poly-A tail. Row six shows the total number of bases in poly-A tails so defined. Row seven shows the total number of nucleotides mapped to mismatching nucleotides in the genome. Row eight shows the total number of inserted nucleotides, defined as unmapped nucleotides that are internal to the mapping. Row nine shows the total number of nucleotides in deletions (genomic nucleotides missing from the clone that do not correspond to introns). Row ten shows the total number of nucleotides in insertions, deletions, or mismatches within 10 basepairs of the splice boundaries. Finally row eleven shows the total number of clones that were considered misoriented. In rows 1 and 11 the percentages indicate the fraction of the total number of nucleotides in all cDNAs, and the total number of cDNAs respectively. In all other rows the percentages indicate the relative change compared to the SPA mappings.
